# Effects of Induced Physical Fatigue on Heart Rate Variability in Healthy Young Adults †

**DOI:** 10.3390/s25175572

**Published:** 2025-09-06

**Authors:** Pei-Chun Kao, David J. Cornell

**Affiliations:** 1 Department of Physical Therapy and Kinesiology, University of Massachusetts Lowell, Lowell, MA 01854, USA; david_cornell@uml.edu; 2 New England Robotics Validation and Experimentation (NERVE) Center, University of Massachusetts Lowell, Lowell, MA 01852, USA; 3 Health Assessment Laboratory, University of Massachusetts Lowell, Lowell, MA 01851, USA

**Keywords:** heart rate variability, physical fatigue, endurance performance, fatigue monitoring, autonomic regulation

## Abstract

Detecting physical fatigue can help prevent overexertion. While typically defined at the muscle level, systemic fatigue remains less clear. Heart rate variability (HRV) reflects autonomic adaptability to physical stressors and may provide insight into fatigue-related responses. This study investigated the impact of physical fatigue on HRV and its correlation with endurance performance. Twenty participants (9 F, 11 M; 23.4 ± 5.0 y) walked on the treadmill at 1.25 m/s with progressively increased incline. HRV metrics were derived from baseline standing (STAND), pre-fatigued (PRE) and post-fatigued walking (POST). Time-domain HRV measures (lnTRI and lnTINN) were significantly reduced at POST compared to PRE or STAND (*p* < 0.05). Non-linear measures (DFA-α1, lnApEn, and lnSampEn) decreased at POST, while lnPoincaré SD2/SD1 increased. Normalized frequency-domain measures showed no condition effects. Baseline non-linear measures (lnApEn, lnSampEn, lnPoincaré SD2/SD1), normalized frequency measures and Total Power were significantly correlated with total fatiguing duration. Significant reductions in HRV and irregularity were observed post-fatigue. Greater baseline variability, irregularity, and high-frequency band power, reflecting parasympathetic activity, were associated with better endurance performance. Time-domain and non-linear measures were more sensitive to fatigue, whereas frequency-domain measures remain useful for identifying associations with endurance. The findings highlight HRV features that could enhance wearable sensing for fatigue and performance.

## 1. Introduction

The ability to detect a person’s physical fatigue status and predict their endurance during prolonged, strenuous activities is crucial for preventing over-exertion and enhancing performance and safety. Physical fatigue is typically defined as a temporary decline in a muscle’s maximal force-generating or power-generating ability following physical activities or tasks [1,2]. It can result from both central and peripheral factors, including reduced neural drive to muscles, metabolic changes, and energy depletion [3,4]. Additionally, physical fatigue is considered a self-reported symptom influenced by both perceived and performance fatigability, modulated by various factors such as homeostasis, psychological state, muscle contractile function and activation [5]. Despite its multifaceted nature and varying definitions, accurately measuring physical fatigue and establishing methods for its detection and monitoring remain significant challenges. Addressing these challenges is essential for developing effective strategies to enhance the performance and safety of individuals engaging in strenuous activities.

These concerns are particularly relevant for physically demanding professions. Occupational groups such as military personnel, firefighters, law enforcement officers, and emergency medical responders are routinely required to perform strenuous tasks while carrying heavy equipment [6,7,8]. The issue of overburdening soldiers has become a significant concern due to the increasing physical demands of military tasks. The load carriage that soldiers are required to carry has steadily risen throughout history [9,10]. Army foot marches while carrying heavy body armor and backpack loads has been shown to cause muscle fatigue in lower extremity, back, and respiratory muscles, which may lead to an increased risk of musculoskeletal injuries [11] and expiratory flow limitation [12]. To mitigate these risks, preventative strategies such as standardized exercise programming have proven effective in enhancing soldiers’ physical fitness levels, preparing them for the physical demands of their duties [13,14,15]. Additionally, the implementation of pre-accession physical fitness screening helps identify low-fit trainees, allowing for targeted interventions to reduce training-related injuries [16,17]. However, these injury prevention initiatives do not directly address over-exertion during both combat and non-combat, military training.

To address this monitoring gap, substantial research has been devoted to exploring the relationship between physical fatigue and heart rate variability (HRV). HRV, which measures fluctuations in successive heartbeats, or beat-to-beat intervals, could provide valuable information on fatigue-related changes and may be particularly useful for longitudinal monitoring during strenuous activities [18,19]. These fluctuations provide insights into the balance between the parasympathetic and sympathetic activities of the autonomic nervous system (ANS), with higher HRV indicating greater parasympathetic activity or reduced sympathetic activity [20,21]. It has also been shown that HRV is associated with the adaptability of the ANS to both physical and psychological stressors [22,23], as well as various physiological states, including aging and diseases [24,25,26]. Significant inverse associations have been observed between subjective ratings of fatigue and both time-domain and frequency-domain HRV measures [19,27], highlighting the potential of HRV as a marker for physical fatigue. However, previous studies primarily relied on self-reported methods, such as subjective exertion rating scales or questionnaires, to assess fatigue status [19,27,28,29]. While these methods can indicate an individual’s perception of fatigue, they may not accurately reflect the actual extent of physical fatigue.

The objectives of this study were twofold: (1) to investigate the impact of physical fatigue on HRV measures, and (2) to identify HRV metrics that are associated with endurance performance. To achieve a more robust assessment of fatigue status, we employed a multi-criterion approach by combining subjective fatigue assessments (e.g., perceived exertion) with an objective functional performance criterion (i.e., vertical jump height) to verify the onset of fatigue. This strategy allowed us to confirm that participants reached a fatigued state involving both cardiovascular and neuromuscular components, a step that many previous studies have not independently validated. We examined changes in HRV data across three conditions: baseline standing, baseline walking, and post-fatigued walking. Physical fatigue was induced via an incline treadmill walking protocol [30]. To comprehensively characterize autonomic modulation, we analyzed a full suite of HRV metrics spanning time-domain, frequency-domain, and non-linear domains. The inclusion of both standing and walking baselines enabled us to distinguish the effects of fatigue from those of physical activity intensity alone, thus improving the specificity of our findings. We hypothesized that HRV would decrease during post-fatigued walking compared to baseline standing or walking condition. For the second objective, we examined whether baseline HRV measures were associated with individual differences in endurance performance, quantified by the total duration participants were able to sustain the incline treadmill walking task.

## 2. Methods

### 2.1. Participants

Twenty healthy young participants (9 females: age: 24.0 ± 7.0 years, body height: 1.61 ± 0.07 m, body mass: 62.3 ± 8.6 kg; 11 males: age: 22.8 ± 2.8 years, body height: 1.80 ± 0.09 m, body mass: 86.9 ± 12.1 kg, mean ± STD) gave written informed consent to participate in the study. This study complied with the tenets of the Declaration of Helsinki and was approved by the Institutional Review Board (IRB) of the University of Massachusetts Lowell (#20-057). The Inclusion criteria were that healthy young participants had to be free of illness or any medical condition that may affect the safety and capability of performing exercises, had no history of neurological problems or major leg injuries, and had no pain in the legs or spine that limit walking.

### 2.2. Experimental Procedures

**Figure 1:**
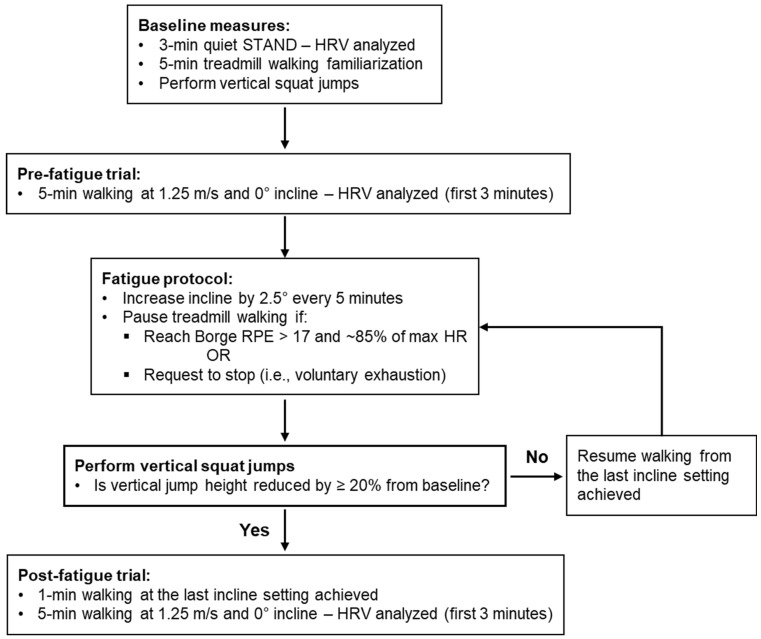
Experimental protocol.

Participants first underwent a 3-min period of quiet standing (Figure 1). They were then given time to familiarize themselves with walking on the treadmill (M-Gait, Motek, The Netherlands) before the testing. Following this, participants were instructed to perform ~3 vertical squat, non-countermovement jumps with one arm crossed over the chest and using the other arm to reach and tap the vanes of Vertec vertical jump tester (JumpUSA, Sunnyvale, CA, USA). Participants’ maximal jump height (inch) among all attempts was recorded [31]. After the jumps, participants walked on the treadmill at 1.25 m/s and 0° of incline for 5 min, both before and after the physical fatiguing protocol (i.e., pre- and post-fatigue trials, respectively). The procedures to induce physical fatigue were detailed in Kao et al. (2023) [30]. Briefly, the inclination angle of the treadmill was increased by 2.5° every five minutes until participants reported a Borg rating of perceived exertion (RPE) > 17/20 and reached ~85% of their maximum age-predicted heart rate or they expressed to pause the protocol due to fatigue (i.e., voluntary exhaustion). At this point, we paused the physical fatiguing protocol and asked participants to perform another 2–3 squat jumps. If participants’ vertical jump height decreased by ≥20%, the physical fatiguing protocol was terminated. Otherwise, the physical fatiguing protocol resumed from the last incline setting achieved and continued until the pausing criteria were met again. Once all criteria were met, participants were promptly returned to the treadmill and walked for one minute at the final incline setting before proceeding to the post-fatigued walking trial at 0° incline. This one-minute walk at the steepest incline achieved was intended to help preserve the fatigued state and minimize potential physiological recovery during the brief transition. The total fatiguing duration was recorded in minutes and represents the cumulative duration of treadmill walking required to induce physical fatigue, excluding pre- and post-fatigue walking trials, as well as the brief vertical jump assessments and rest periods between fatiguing bouts [30].

### 2.3. Data Collection and Analysis

Raw beat-to-beat RR interval data, measured as the time intervals between successive R-peaks of the electrocardiogram (ECG), were recorded using a chest harness (Equivital EQ02 LifeMonitor, New York, NY, USA) with the raw ECG waveform collected at 256 Hz. HRV analyses were conducted on beat-to-beat RR interval data exported from the Equivital system and processed using Kubios HRV Standard ver. 3.5.0 (Kubios Ltd., Kuopio, Finland). In Kubios, we applied the default preprocessing settings for HRV analysis [32]. Specifically, we used the Smoothness Priors detrending method (λ = 500) for time- and frequency-domain measures. For non-linear HRV measures, we did not apply detrending, as detrending has been reported to distort short-term non-linear HRV metrics while having minimal impact on linear measures [33,34]. To ensure data integrity, we visually inspected all RR time series to identify and exclude outliers.

**Figure 2:**
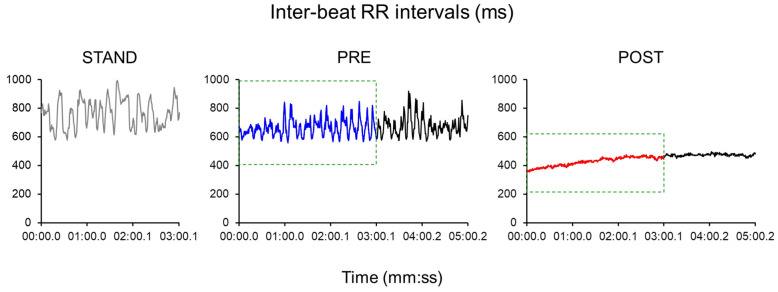
Example of the inter-beat RR interval data series collected from a representative participant across conditions (STAND, PRE, and POST). Only the first 3 min of the PRE and POST walking trials were included in the analysis.

Outcome measures were derived for baseline standing (STAND), pre-fatigued walking (PRE), and post-fatigued walking (POST) trials. Respiratory rate data were also recorded using the Equivital system. To ensure consistency across conditions, only the first 3 min of data were analyzed for each trial, including PRE and POST walking, to match the 3-min standing trial duration (Figure 2). Three-minute recordings have been shown to provide reliable estimates of time-, frequency-, and non-linear domain measures comparable to the standard 5-min recordings under steady-state conditions [35,36,37]. For time-domain HRV measures, we derived the root mean square of successive RR interval differences (RMSSD) as well as the triangular index (TRI) and triangular interpolation (TINN) of RR intervals based on the geometric features [32]. TRI is the integral of the RR interval histogram divided by the height of the histogram while TINN represents the baseline width of the RR interval histogram. Larger values of these time-domain HRV measures indicate greater HRV.

For frequency-domain measures, we examined the power spectrum of RR interval data. We derived the total power, percentage of low-frequency (pLF) band power to the total power (low frequency: 0.04–0.15 Hz), percentage of high-frequency (pHF) band power to the total power (high frequency: 0.15–0.40 Hz), and the ratio between low-frequency and high-frequency band powers (LF/HF) [32]. Total power refers to the sum of all power spectral density components, reflecting the total variance in heart rate pattern. The LF power band may not solely reflect sympathetic activity due to influences from multiple physiological systems, including respiratory influences, baroreceptor activity, and other autonomic regulatory mechanisms. In contrast, HF power band is a more direct indicator of parasympathetic activity [21]. Additionally, the ratio of LF to HF (LF/HF) is commonly used to represent the balance between sympathetic and parasympathetic activity, with a low LF/HF ratio indicating parasympathetic dominance.

We used detrended fluctuation analysis (DFA), Poincaré plot, approximate entropy, and sample entropy to derived non-linear HRV measures [32]. DFA measures correlations within RR-interval data across different time segments. We specifically examined short-term fluctuation using the short-term scaling exponent (DFA-α1), which are characterized by the scaling exponent corresponding to the slopes of regression lines on log-log plots that relate fluctuations of the integrated and detrended RR-interval data to segment lengths of 4–16 samples [32]. Short-term DFA (DFA-α1) has been suggested to be more related to parasympathetic modulation [38,39]. A decrease in DFA-α1 indicates tighter control over heart rate, reflecting reduced HRV [40].

Approximate entropy (ApEn) quantifies the regularity of RR-interval data by measuring the logarithmic likelihood that runs of patterns close to each other will remain close in subsequent incremental comparisons [41]. A smaller ApEn value indicates a higher likelihood of patterns remaining consistent, which reflects a greater degree of regularity and predictability, and thus, indicating reduced HRV. Sample entropy (SampEn) is a modified version of ApEn that eliminates the biased self-counting and is less influenced by the length of the data series [42]. Both ApEn and SampEn were examined to provide insights into the regularity of heart rate dynamics. The Poincaré plot, a two-dimensional reconstruction of RR interval phase-space, illustrates the temporal correlation between consecutive RR intervals by plotting each RR interval data point against the preceding one [32]. To characterize the shape of the Poincaré plot, the RR interval data points were fitted into an ellipse oriented along the line of identify. The width (SD1) and length (SD2) of this ellipse were determined by the standard deviations of the points perpendicular and parallel to the line of identity, respectively. SD1 measures short-term HRV, reflecting parasympathetic modulation, while SD2 quantifies long-term HRV, representing the modulation of both parasympathetic and sympathetic systems [43]. The ratio of SD2 to SD1 (SD2/SD1) indicates the balance between long- and short-term HRV, with a larger ratio of SD2/SD1 indicating increased sympathetic activity. It has been shown that the SD2/SD1 ratio correlates with the LF/HF ratio [44].

### 2.4. Statistical Analysis

We conducted Shapiro–Wilk tests to assess the normality of outcome measures. Except for DFA-α1, heart rate, and respiratory rate, we found that all other measures were non-normally distributed, and thus, underwent natural log (ln) transformation to correct for the observed skewness (Table 1 and Table 2). We used repeated measures ANOVAs to test for differences in HRV measures across the three conditions (STAND, PRE, and POST). We used Tukey Honestly Significant Difference (THSD) post hoc tests for pair-wise comparisons if significant ANOVA effects were detected.

Time-domain HRV measures and Poincaré-plot measures (SD1, SD2) are mathematically tied to average RR [37]. In addition, entropy-based non-linear measures (ApEn and SampEn) are known to be sensitive to changes in average RR [37,45]. Moreover, respiratory rate is known to influence high-frequency (HF) power and LH/HF ratio [46]. Therefore, the natural log of RR interval (lnRR) was included as a covariate for time-domain HRV parameters, the Poincaré SD2/SD1 ratio, and entropy-based non-linear measures, while respiratory rate was included as a covariate for frequency-domain HRV parameters.

We calculated effect sizes using partial eta squared (η^2^) for ANOVA main effects and Cohen’s d for pairwise post hoc comparisons [47,48]. Partial η^2^ values were interpreted as: 0.02 “small” effect, 0.13 “medium” effect and 0.26 “large” effect [47,48]. Cohen’s d values were interpreted as: 0.2 “small” effect, 0.5 “medium” effect and 0.8 “large” effect [48]. Pearson’s correlations were used to investigate the association between the total fatiguing duration and HRV measures at STAND and PRE. For time-domain HRV parameters, the Poincaré SD2/SD1 ratio, and entropy measures, partial correlations were performed controlling for lnRR. For frequency-domain HRV parameters, partial correlations were performed controlling for respiratory rate. We set the significance level at 0.05. All statistical analyses were performed in JMP version 13 (SAS institute Inc., Cary, NC, USA).

## 3. Results

All participants ultimately met all aspects the fatigue criteria, including a RPE > 17/20, ~85% of their maximum age-predicted heart rate, and a ≥20% reduction in vertical jump height. Although voluntary exhaustion was included as a safety-based stopping criterion in the approved IRB protocol, no participants chose to stop before reaching the physiological or performance-based thresholds. Participants showed a 22.8 ± 2.9% reduction in maximum vertical jump height following the fatiguing protocol (baseline: 42.2 ± 8.8 cm; post-fatigue: 32.6 ± 6.8 cm), confirming that they met the performance-based fatigue criterion.

### 3.1. Condition Effects on Heart Rate Variability (HRV) Measures

**Figure 3:**
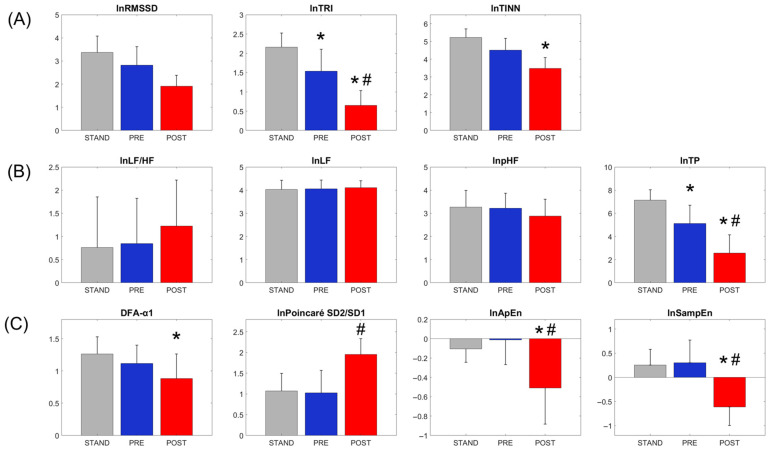
Heart rate variability (HRV) measures across conditions (STAND, PRE, and POST): (**A**) time-domain, (**B**) frequency-domain, and (**C**) non-linear metrics. Error bars represent 1 STD. * indicates significant difference compared to STAND and # indicates significant difference between POST and PRE (THSD, *p* < 0.05), based on adjusted models where applicable (i.e., models including lnRR or breathing rate as covariates, except for DFA-α1).

Significant condition effects were found for inter-beat RR interval (RR), heart rate, and respiratory rate (Table 1). For time-domain HRV measures (Table 3), the natural log values of RMSSD (lnRMSSD), TRI (lnTRI), and TINN (lnTINN) were significantly reduced at POST compared to both PRE and STAND (THSD, *p* < 0.05) (Figure 3). Additionally, these time-domain measures also showed lower values at PRE compared to Stand (THSD, *p* < 0.05). After adjusting for lnRR as a covariate, lnRMSSD no longer showed a significant condition effect, lnTRI maintained the same patterns of condition effects as in the unadjusted model, and lnTINN remained significantly reduced at POST compared to STAND. Regarding frequency-domain HRV measures, there were no significant condition effects on the natural log values of low-frequency band power percentage (lnpLF), high-frequency band power percentage (lnpHF), and the ratio between low-frequency and high-frequency band powers (lnLF/HF) (ANOVA, *p* > 0.05). However, the natural log value of Total power (lnTP) was significantly reduced at POST compared to PRE or STAND, also had lower values at PRE compared to STAND (THSD, *p* < 0.05). For non-linear HRV measures, the short-term scaling exponent of detrended fluctuation (DFA-α1) was significantly reduced at POST compared to Stand. The natural log values of approximate entropy (lnApEn) and sample entropy (lnSampEn) were significantly reduced at POST compared to both PRE and STAND (THSD, *p* < 0.05). Additionally, the natural log value of the Poincaré SD2/SD1 ratio (lnPoincaré SD2/SD1) was significantly increased at POST compared to PRE (THSD, *p* < 0.05).

### 3.2. Correlations Between the Total Fatiguing Duration and Baseline HRV Measures

lnRR and heart rate at both STAND and PRE were significantly correlated with total fatiguing duration (*p* < 0.05) (Table 3). For time-domain HRV measures, no significant correlations with total fatiguing duration were observed after controlling for lnRR. For frequency-domain HRV measures, lnLH/HF, lnpLF, lnpHF at STAND, as well as lnTP at PRE were significantly correlated with total fatiguing duration. lnLH/HF and lnpLF showed negative correlations, while lnpHF and lnTP showed positive correlations. Except for DFA-α1, all other non-linear HRV measures were significantly correlated with total fatiguing duration. Specifically, lnPoincaré SD2/SD1, lnApEn and lnSampEn at PRE were significantly correlated with total fatiguing time. lnPoincaré SD2/SD1 showed a negative correlation, while lnApEn and lnSampEn showed positive correlations.

## 4. Discussion

The current study investigated the impact of physical fatigue on HRV and its association with endurance performance. Consistent with our hypothesis, we found that physical fatigue led to significant reductions in HRV, particularly as demonstrated by time-domain and non-linear parameters. For time-domain HRV measures, significant reductions were observed in lnRMSSD, lnTRI, and lnTINN during post-fatigued walking compared to both baseline walking and standing conditions. In addition, the values of these time-domain measures were also lower during baseline walking compared to baseline standing, indicating their sensitivity to changes in physical activity intensity independent of fatigue, consistent with previous reports of reduced time-domain HRV as exercise intensity increases [49,50]. Although HRV was lower in the POST condition, the absolute magnitude of beat-to-beat variability (e.g., RMSSD ≈ 7.7 ms) remained above the ~4 ms resolution limit of our 256 Hz sampling rate, supporting the validity of HRV analyses even in this reduced variability state. However, when controlling for mean inter-beat RR intervals (lnRR), lnRMSSD no longer showed a significant condition effect, suggesting that its changes may partly reflect shifts in heart rate rather than autonomic modulation alone. In contrast, lnTRI and lnTINN remained sensitive to condition effects even after adjusting for lnRR, highlighting their robustness as fatigue indicators.

For frequency-domain HRV measures, we found that lnTP was significantly reduced during post-fatigued walking compared to both baseline walking and standing, and was also lower during baseline walking compared to baseline standing. These results align with previous studies demonstrating that total power, along with the power of individual frequency components, decreases with increasing exercise intensity or following exhaustive exercise [50,51]. In contrast, we observed no significant condition effects on lnpLF, lnpHF, or lnLF/HF. Conflicting findings for these normalized and ratio measures of LF and HF have been reported in the literature [50]. For example, some studies suggest that the LF/HF ratio decreases with higher exercise intensity, while others report an increase in this ratio with greater exercise intensity [50]. Overall, our findings suggest that total power, which reflects the overall variance in heart rate patterns, decreases with greater physical activity intensity and the presence of physical fatigue whereas the ratios or relative contributions of individual frequency components remain unchanged regardless of activity intensity or physical fatigue status. It should also be recognized, however, that exercise characteristics beyond intensity, such as duration, recovery, and modality, may also influence HRV responses and contribute to the divergent findings reported in the literature [50,52,53].

For non-linear HRV measures, significant changes were observed during post-fatigued walking compared to baseline walking and/or standing. Specifically, DFA-α1 was significantly reduced at POST compared to STAND, reflecting a loss of fractal-like correlation properties in heart rate dynamics and is often associated with decreased autonomic complexity following fatigue [20,40]. Similarly, lnApEn, and lnSampEn, which quantify the irregularity of inter-beat RR intervals, were significantly reduced at POST compared to both baseline walking and standing, indicating increased regularity in autonomic modulation with fatigue [20]. In addition, the lnPoincaré SD2/SD1 ratio significantly increased at POST compared to baseline walking. Wang et al. (2024) reported that both short-term HRV (SD1) and long-term HRV (SD2) were reduced following exhaustive exercise, resulting in a significant increase in the Poincare SD2/SD1 ratio [51], consistent with our findings. Since SD1 primarily reflects parasympathetic modulation and SD2 represents combined parasympathetic and sympathetic influences [43], these results suggest a significant inhibition of parasympathetic nervous activity after exhaustive exercise. Taken together, these findings indicate that heart rate signals became more tightly controlled following physical fatigue [40,41,43]. In contrast, none of these non-linear measures differ significantly between baseline walking and baseline standing, indicating that these measures are relatively insensitive to changes in physical activity intensity alone. Notably, previous studies have also reported reductions in DFA-α1 and entropy measures [40,54,55,56] and increases in the lnPoincaré SD2/SD1 ratio [51] in response to both increasing exercise intensity and physical fatigue. Somewhat different from earlier findings, which suggest sensitivity to both exercise intensity and exhaustion, our results demonstrated that non-linear HRV measures are more specific to detecting physical fatigue status, as their values seem to be less influenced by physical activity intensity alone (e.g., walking versus quiet standing). This distinction highlights their potential utilization in isolating fatigue effects from those related to activity intensity, making them promising markers for monitoring fatigue.

Our previous study examined the effects of physical fatigue on walking mechanics and energetics, demonstrating that fatigue negatively affected walking economy, reduced mechanical work efficiency, and altered the mechanical work distribution among lower-limb joints, with a shift in positive work contribution from the ankle to the knee joint [30]. Combined with the current findings, these results highlight the extensive impact of physical fatigue on both cardiovascular and biomechanical systems, as well as on cardiac autonomic regulation. While the distal-to-proximal redistribution of joint mechanical work has been proposed as a potential indicator of physical fatigue during walking or running [30,57], the need for extensive sensors and equipment to collect data limits its practicality for real-world applications in detecting physical fatigue status. In contrast, HRV metrics, particularly non-linear measures such as DFA-α1 and entropy, offer a more accessible alternative for monitoring fatigue. These HRV metrics can detect physical fatigue and are less influenced by the activity level difference between standing and walking, making them well suited for timely assessments. This distinction underscores the potential utility of HRV as a valuable and portable tool for fatigue detection, since it can be measured outside of laboratory settings where biomechanical assessments may not be practical. Although the present study was conducted under controlled conditions, these findings highlight the translational potential of HRV for monitoring fatigue in applied and occupational contexts (e.g., ruck marches). Future work should extend this approach to field-based settings and examine fatigue across longer time frames, such as the cumulative effects of a full workday.

The second objective of this study was to identify baseline HRV measures that are associated with endurance performance. We examined the relationship between baseline HRV metrics and participants’ endurance performance, quantified by total fatiguing duration. Our results showed that lnRR and mean heart rate at both baseline standing and walking were significantly correlated with total fatiguing duration, emphasizing their association with individual differences in endurance performance. After adjusting for lnRR, time-domain HRV measures no longer showed significant associations with endurance performance. Although the natural log values of TRI and TINN, as well as DFA-α1, were able to detect physical fatigue, no significant correlations were found between these metrics at baseline and total fatiguing duration. In contrast, while normalized frequency-domain HRV metrics (i.e., pLF, pHF, and the LH/HF ratio) did not detect physical fatigue, their values during baseline standing showed significant correlations with total fatiguing duration. Specifically, lnpLF and lnLH/HF exhibited negative correlations, whereas lnpHF demonstrated a positive correlation. Despite these normalized and ratio-based measures of LF and HF not detecting condition effects, their associations with endurance performance offer valuable insights [58]. Since the high-frequency power band is commonly interpreted as an indicator of parasympathetic activity, and the LF/HF ratio is widely used to reflect the balance between sympathetic and parasympathetic modulation [21], our findings suggest that individuals with greater parasympathetic modulation, reflected by higher lnpHF and lower lnpLF and lnLH/HF values, tend to demonstrate enhanced endurance performance. Notably, non-linear HRV measures, with the exception of DFA-α1, at baseline walking (PRE) were also significantly correlated with total fatiguing duration. lnPoincaré SD2/SD1 was negatively correlated, while lnApEn and lnSampEn were positively correlated with fatiguing time, suggesting that non-linear HRV metrics captured during baseline walking may better reflect endurance capacity.

While our study did not assess training adaptations, previous research has shown that exercise training enhances parasympathetic modulation, evidenced by increased HF power or reduced LF to HF ratio, and greater overall HRV responses [59,60,61,62], suggesting that the relationship between HRV and endurance capacity may be bidirectional. At the same time, the correlations observed in the current study should be interpreted with caution, as they do not imply causality. Other factors such as aerobic fitness, physical activity history, or sex-based physiological differences may have also influenced the associations observed between baseline HRV and total fatiguing duration [63,64]. In addition, the relatively small sample size (n = 20) limits the generalizability of these findings. The exploratory nature of these analyses further suggests that the results should be viewed as preliminary rather than definitive evidence of a predictive role of HRV. Nonetheless, the consistency of our observations with prior work strengthens the plausibility of these associations. Consequently, the novelty of this result lies not in establishing HRV as a conclusive predictor of endurance capacity, but in identifying promising candidate metrics, particularly baseline non-linear and frequency-domain measures, that warrant further investigation in larger and more diverse populations.

Our findings highlight the potential of using HRV to provide insight into fatigue-related autonomic responses and their association with endurance performance. Although no consensus exists on the best HRV metrics for predicting endurance performance, higher baseline HRV is generally associated with better performance, reflecting greater capacity for physical exertion and recovery [50,61]. Endurance athletes also tend to exhibit higher resting HRV compared to less active individuals [23,65], suggesting that HRV could serve as a valuable indicator of overall fitness levels in tactical populations. It should be noted, however, that HRV indices alone may not fully capture all aspects of readiness or performance, as they primarily reflect cardiac autonomic activity. Combining HRV measures with other indices, such as submaximal heart rate or neuromuscular performance tests, may provide a more comprehensive assessment of fatigue and performance [66]. Future studies should further investigate relationships between HRV and endurance performance across different fatiguing activities (e.g., whole-body versus localized muscular fatigue [67]) and among tactical athletes with varying fitness levels. In addition, it is important to consider the potential of HRV for long-term fatigue monitoring, beyond single or daily measurements, to capture cumulative effects over extended periods.

The current study specifically examined the effects of fatigue on HRV during level walking at a matched workload (same treadmill speed and grade before and after fatigue). While this approach helped isolate fatigue effects from exercise intensity, understanding how fatigue progresses and accumulates across increasing workloads warrants further investigation. It is also important to note that while our results show certain HRV measures (e.g., non-linear indices) change with fatigue and relate to endurance performance, the current study was not designed to define specific HRV thresholds for identifying a fatigued state. Larger, targeted studies are needed to determine whether cutoff values can be established. By identifying the most relevant HRV metrics and integrating them with complementary measures, it may be possible to refine fatigue monitoring and develop personalized physical training and recovery protocols, thereby enhancing performance and reducing the risk of over-exertion in military and other physically demanding professions.

A potential limitation of this study is the lack of control over physiological and behavioral factors that can influence HRV. In addition to physical fatigue, HRV can be influenced by various factors, such as stress, hydration, sleep quality, nutritional status, circadian rhythms, and medication use, which were not recorded and monitored in this study and may have affected HRV measurements to some extent [68,69,70,71]. While participants were reminded to consume water during breaks to reduce the risk of dehydration, we did not systematically control the time of day for testing or record participants’ sleep quality or fasting status. Testing sessions occurred at various times between approximately 9 am and 5 pm, which may have introduced some variability among participants. Similarly, we did not explicitly monitor participants’ medication use; however, our inclusion criteria required that participants be healthy young adults without known medical conditions. Additionally, because the study was conducted exclusively with healthy young adults, the findings may not be generalizable to older adults, clinical populations, or individuals with lower fitness levels, and thus, follow-up research within tactical populations is needed as well.

Another consideration is the potential influence of anthropometric differences or sex-based differences on HRV [63,72]. Body size and composition may indirectly affect HRV through mechanisms such as cardiorespiratory fitness, resting heart rate, and autonomic tone. In the current study, both male and female participants were included, and they differed substantially in height and weight, which could introduce variability in HRV responses. However, because we employed a within-subject design that focused on condition-based changes (i.e., baseline standing, pre-fatigued walking, and post-fatigued walking) within each participant, the potential impact of between-subject variability, including anthropometric and other uncontrolled physiological factors, is likely reduced. Moreover, although vertical jump height provides a non-invasive and practical method for objectively assessing fatigue, incorporating additional physiological or biochemical markers, such as blood lactate levels or muscle oxygenation, will provide a more comprehensive evaluation of physiological fatigue.

## 5. Conclusions

The current study investigated the effects of physical fatigue on HRV measures and their associations with endurance performance. Significant reductions in HRV or irregularity were observed during post-fatigued walking in time-domain and non-linear measures, while normalized frequency-domain measures showed no significant condition effects. Our findings also indicate that having greater variability, irregularity, and high-frequency band power in HR signals, reflecting increased parasympathetic activity at baseline, is associated with better endurance performance. These results suggest that time-domain and non-linear parameters are more sensitive to detecting physical fatigue, while normalized frequency parameters remain valuable for identifying associations with endurance performance. Incorporating HRV monitoring could help optimize performance and prevent over-exertion in physically demanding professions.

## Figures and Tables

**Table 1:** Raw cardiorespiratory data (mean ± STD) during baseline standing (STAND), walking at 1.25 m/s before (PRE) and after (POST) the physical fatiguing protocol.

Raw Cardiorespiratory Parameters	Conditions			Condition Effect ANOVA, *p*-Value (Power; Partial η^2^)	Post Hoc Comparison (THSD, *p* < 0.05; Cohen’s d)
	STAND	PRE	POST		
Inter-beat RR interval (**RR**, ms)	726.6 ± 175.4	590.5 ± 103.3	423.7 ± 62.8	—	—
Natural log of RR interval (**lnRR**)	6.56 ± 0.22	6.37 ± 0.17 *	6.04 ± 0.14 *^#^	**<0.001** (1.000; 0.902)	POST vs. STAND (d = 5.85) POST vs. PRE (d = 3.66) PRE vs. STAND (d = 2.20)
Heart rate (bpm)	86.6 ± 18.3	104.4 ± 17.3 *	144.1 ± 18.5 *^#^	**<0.001** (1.000; 0.930)	POST vs. STAND (d = 6.95) POST vs. PRE (d = 4.80) PRE vs. STAND (d = 2.15)
Respiratory rate (breaths/min)	17.7 ± 3.5	27.1 ± 5.7 *	33.8 ± 5.6 *^#^	**<0.001** (1.000; 0.817)	POST vs. STAND (d = 2.98) POST vs. PRE (d = 1.57) PRE vs. STAND (d = 0.97)

Note: Inter-beat RR interval (RR) violated normality assumptions and is reported in both original and log-transformed scales. *: significant difference compared to STAND (post hoc, THSD, *p* < 0.05) #: significant difference between POST and PRE (post hoc, THSD, *p* < 0.05). *p*-values less than 0.05 are presented in bold.

**Table 2:** Descriptive Statistics and Statistical Comparisons of Heart Rate Variability (HRV) Parameters Across Conditions (STAND, PRE, and POST).

Heart Rate Variability (HRV) Parameters	Conditions			Condition Effect ANOVA, *p*-Value (Power; Partial η^2^)	Post Hoc Comparison (THSD, *p* < 0.05; Cohen’s d)
	STAND	PRE	POST		
**Time-domain measures**					
Root mean square of successive RR interval differences (RMSSD, ms)	37.7 ± 32.3	22.6 ± 18.5	7.7 ± 4.6	—	—
Natural log of RMSSD (lnRMSSD)—unadjusted	3.37 ± 0.70	2.82 ± 0.79	1.91 ± 0.47	**<0.001** (1.000; 0.704)	POST vs. STAND (d = 2.97) POST vs. PRE (d = 1.85) PRE vs. STAND (d = 1.12)
lnRMSSD—adjusted for lnRR	—	—	—	0.948	—
Triangular index of RR intervals (TRI)	9.3 ± 4.0	5.44 ± 3.24	2.10 ± 1.27	—	—
Natural log of TRI (lnTRI)—unadjusted	2.16 ± 0.36	1.54 ± 0.57	0.65 ± 0.38	**<0.001** (1.000; 0.659)	POST vs. STAND (d = 5.44) POST vs. PRE (d = 3.20) PRE vs. STAND (d = 2.24)
lnTRI—adjusted for lnRR	—	—	—	**0.003** (0.885; 0.282)	POST vs. STAND (d = 3.77) POST vs. PRE (d = 2.15) PRE vs. STAND (d = 1.62)
Triangular interpolation of RR intervals (TINN, ms)	208.0 ± 115.3	110.8 ± 70.2	41.0 ± 39.0	—	—
Natural log of TINN (lnTINN)—unadjusted	5.22 ± 0.48	4.51 ± 0.67	3.48 ± 0.62	**<0.001** (1.000; 0.775)	POST vs. STAND (d = 3.59) POST vs. PRE (d = 2.12) PRE vs. STAND (d = 1.47)
lnTINN—adjusted for lnRR	—	—	—	**0.028** (0.696; 0.126)	POST vs. STAND (d = 1.41)
**Frequency-domain measures**					
Ratio between LF and HF band powers (LF/HF)	3.53 ± 3.29	3.49 ± 3.18	5.28 ± 4.96	—	—
Natural log of the ratio between LF and HF band powers (lnLF/HF)—unadjusted	0.76 ± 1.09	0.84 ± 0.98	1.23 ± 0.99	0.314	—
lnLF/HF—adjusted for breathing rate	—	—	—	0.790	—
Percentage of low-frequency band power to the total power (pLF, %)	60.0 ± 20.5	61.2 ± 18.0	63.2 ± 17.4	—	—
Natural log of the percentage of low-frequency band power to the total power (lnpLF)—unadjusted	4.03 ± 0.40	4.06 ± 0.37	4.11 ± 0.30	0.781	—
lnpLF—adjusted for breathing rate	—	—	—	0.967	—
Percentage of high-frequency band power to the total power (pHF, %)	32.7 ± 20.2	30.0 ± 17.6	22.4 ± 14.6	—	—
Natural log of the percentage of high-frequency band power to the total power (lnpHF)—unadjusted	3.27 ± 0.72	3.22 ± 0.66	2.88 ± 0.73	0.176	—
lnpHF—adjusted for breathing rate	—	—	—	0.659	—
Total power (T**P**, ms^2^)	1920.6 ± 2143.8	504.4 ± 800.2	61.4 ± 186.3	—	—
Natural log of the Total power (lnTP)—unadjusted	7.13 ± 0.91	5.12 ± 1.57	2.57 ± 1.58	**<0.001** (1.000; 0.859)	POST vs. STAND (d = 4.79) POST vs. PRE (d = 2.68) PRE vs. STAND (d = 2.12)
lnTP—adjusted for breathing rate	—	—	—	**<0.001** (0.999; 0.474)	POST vs. STAND (d = 3.90) POST vs. PRE (d = 2.32) PRE vs. STAND (d = 1.58)
**Non-linear measures**					
Short-term scaling exponent of detrended fluctuation (DFA**-α1**)	1.26 ± 0.26	1.12 ± 0.28	0.88 ± 0.38	**0.001** (0.939; 0.295)	POST vs. STAND (d = 1.25)
Poincaré SD2/SD1 ratio	3.16 ± 1.24	3.26 ± 2.27	7.56 ± 2.85	—	—
Natural log of Poincaré SD2/SD1 (lnPoincaré SD2/SD1)—unadjusted	1.07 ± 0.43	1.02 ± 0.54	1.96 ± 0.38	**<0.001** (1.000; 0.576)	POST vs. STAND (d = 1.91) POST vs. PRE (d = 2.02)
lnPoincaré SD2/SD1—adjusted for lnRR	—	—	—	**0.002** (0.926; 0.208)	POST vs. PRE (d = 1.43)
Approximate entropy (ApEn)	0.91 ± 0.12	1.01 ± 0.19	0.64 ± 0.23	—	—
Natural log of Approximate entropy (lnApEn)—unadjusted	−0.10 ± 0.14	−0.01 ± 0.26	−0.51 ± 0.38	**<0.001** (0.999; 0.471)	POST vs. STAND (d = 1.73) POST vs. PRE (d = 1.41)
lnApEn—adjusted for lnRR	—	—	—	**<0.001** (0.989; 0.283)	POST vs. STAND (d = 1.86) POST vs. PRE (d = 1.57)
Sample entropy (SampEn)	1.35 ± 0.39	1.46 ± 0.45	0.58 ± 0.23	—	—
Natural log of Sample entropy (lnSampEn)—unadjusted	0.25 ± 0.33	0.30 ± 0.47	−0.61 ± 0.39	**<0.001** (1.000; 0.621)	POST vs. STAND (d = 2.22) POST vs. PRE (d = 2.10)
lnSampEn—adjusted for lnRR	—	—	—	**<0.001** (0.989; 0.285)	POST vs. STAND (d = 1.85) POST vs. PRE (d = 1.42)

Note: All HRV parameters are presented in both original and log-transformed scales, except DFA-α1, which did not violate normality assumptions and is reported only in its original form. Values are reported as mean ± standard deviation (STD). Unadjusted models reflect repeated-measures ANOVA without covariates. Adjusted models include either the natural log of inter-beat RR interval (lnRR) or respiratory rate as covariates, as specified. *p*-values less than 0.05 are presented in bold.

**Table 3:** Correlations between total fatiguing duration and raw cardiorespiratory parameters or HRV measures at baseline standing (STAND) and pre-fatigued walking (PRE).

Raw Cardiorespiratory Parameters and Heart Rate Variability (HRV) Parameters	Stand		PRE	
	*r*	*p*-Value	*r*	*p*-Value
lnRR	**0.466** ^†^	0.019	**0.417** ^†^	0.034
Heart rate	**−0.434** ^†^	0.028	**−0.414** ^†^	0.039
Respiratory rate	0.012	0.480	−0.240	0.154
**Time-domain measures**				
lnRMSSD—unadjusted	0.293	0.105	**0.390** ^†^	0.045
lnRMSSD—adjusted for lnRR	−0.189	0.220	0.112	0.325
lnTRI—unadjusted	0.175	0.231	**0.416** ^†^	0.034
lnTRI—adjusted for lnRR	−0.229	0.173	0.129	0.299
lnTINN—unadjusted	0.088	0.356	0.241	0.154
lnTINN—adjusted for lnRR	−0.258	0.143	−0.096	0.347
**Frequency-domain measures**				
lnLF/HF—unadjusted	**−0.500** ^†^	0.012	−0.164	0.244
lnLF/HF—adjusted for breathing rate	**−0.436** ^†^	0.031	−0.143	0.279
lnpLF—unadjusted	**−0.488** ^†^	0.015	−0.098	0.341
lnpLF—adjusted for breathing rate	**−0.488** ^†^	0.017	−0.097	0.346
lnpHF—unadjusted	**0.488** ^†^	0.014	0.191	0.210
lnpHF—adjusted for breathing rate	**0.492** ^†^	0.016	0.194	0.213
lnTP—unadjusted	0.011	0.482	**0.423** ^†^	0.031
lnTP—adjusted for breathing rate	0.009	0.485	**0.436** ^†^	0.031
**Non-linear measures**				
DFA-α1	−0.335	0.074	−0.327	0.080
lnPoincaré SD2/SD1—unadjusted	**−0.403** ^†^	0.039	**−0.555** ^†^	0.006
lnPoincaré SD2/SD1—adjusted for lnRR	−0.067	0.393	**−0.485** ^†^	0.018
lnApEn—unadjusted	−0.030	0.449	**0.570** ^†^	0.004
lnApEn—adjusted for lnRR	−0.015	0.476	**0.582** ^†^	0.004
lnSampEn—unadjusted	**0.489** ^†^	0.014	**0.580** ^†^	0.004
lnSampEn—adjusted for lnRR	0.276	0.126	**0.555** ^†^	0.007

Note: Unadjusted values represent standard Pearson correlations with total fatiguing duration. Adjusted values represent partial correlations controlling for either the natural log of inter-beat RR interval (lnRR) or respiratory rate, as indicated. *r*: Pearson correlation coefficient; ^†^: significant correlation between the baseline cardiorespiratory or HRV measures and total fatiguing duration (*p* < 0.05). Significant correlations (*p* < 0.05) are shown in bold.

## Data Availability

Data are contained within the article.
